# Preparation and catalytic evaluation of Au/γ -Al_2_O_3_ nanoparticles for the conversion of 4-nitrophenol to 4-aminophenol by spectrophotometric method

**DOI:** 10.3906/kim-1910-21

**Published:** 2020-04-01

**Authors:** Farhat SAIRA, Naveeda FIRDOUS, Rumana QURESHI, Ayesha IHSAN

**Affiliations:** 1 Nanoscience and Technical Division, National Centre for Physics (NCP), Shahdra Valley Rd, Islamabad Pakistan; 2 Department of Chemistry, Quad-i-Azam University, Islamabad Pakistan; 3 National Institute of Biotechnology and Genetic Engineering (NIBGE), Faisalabad Pakistan

**Keywords:** Gold catalyst, γ -Al_2_O_3_, UV-visible spectroscopy, 4-Nitrophenol

## Abstract

A set of catalysts having gold nanoparticles deposited on γ -Al_2_O_3_ ( Au/ γ -Al_2_O_3_) with lowest effective amount of gold content were prepared by successive impregnation and hydrogen reduction method. The structural features of prepared catalysts were analysed by X-ray diffraction (XRD), N2 physisorption, scanning electron microscopy (SEM), and Fourier transform infrared (FTIR). The catalytic activity was evaluated for the reduction of an organic pollutant 4-nitrophenol (4NP) to 4-aminophenol (4AP) by spectrophotometric analysis. Supported catalyst presented excellent catalytic ability to convert 4NP to 4AP in the presence of sodium borohydride (SBH) due to synergistic effect of Au NPs and mesoporous γ -Al_2_O_3_ support. The reduction reaction was also performed at a range of temperatures to calculate kinetic parameters. The development of highly stable Au/γ -Al_2_O_3_ catalysts with lowest noble metal content and recyclability made the process cost effective and may promote their applications in various fields including removal of organic pollutants in industrial waste water and high-temperature gas-phase reactions.

## 1. Introduction

Supported gold catalysts are currently being used to catalyse various reactions having environmental and industrial significance. The incredible catalytic activity of supported gold nanoparticles (NPs) was first identified by Haruta et al. [1]. Since then, gold NPs have been used as a catalyst in many reactions involving electron transfer process [2-6].

Industrially important catalytic processes mainly require stable supported metal or metal oxide catalysts [7]. The advantages of supported metal catalysts are the uniform metal distribution, no agglomeration, and deposition of less metal content on the support, easy recovery of expensive noble metal, multiple uses of the catalysts, and cost economics [8]. The catalytic performance of a metal deposited on a support depends on the properties of support as well as active metal [9,10]. The support can play a crucial role in understanding the catalytic phenomenon including diffusion of reactants [11,12], metal-support interactions [13,14], hydrophilic character of catalyst [15] etc. Gold NPs have been supported on multiple supports including silica, titania, mixed oxides, zeolites, boehmite, and alumina [16–20]. The most commonly used catalytic support is γ -Al_2_O_3_ due to its high surface area and porosity, optimum mechanical strength, and uniform pore size distribution [21]. Typically, impregnation method is employed for the preparation of supported catalysts. It is based on soaking the support into metal salt solution followed by evaporation and metal reduction. A surfeit of reports on the catalytic activity of supported gold NPs has explored the redox behaviour of Au NPs [12–13], acid–base properties of gas-phase gold, and gold-oxide [14–16]. To date several theories have been put forward to explore an extraordinary catalytic activity of supported gold NPs; nonetheless these theories are somewhat contentious [22–25]. The outcomes of these theories showed that the size of gold NPs, choice of support, and method of preparation played an important role in delivering a highly efficient catalyst [26].

Pal et al. for the first time introduced catalytic reduction of 4-nitrophenol (4-NP) to 4-aminophenol (4-AP) [27–30]. This reaction takes place in aqueous system on the surface of a catalyst. Reduction of this organic pollutant can be conveniently monitored by decrease of strong UV-visible absorption spectra of 4-nitrophenolate anion at 400 nm [31, 32]. Au nanoparticles supported on Al_2_O_3_ and SiO2 are reported to be thermally much more stable than Au nanoparticles on TiO2 . For example, thermal treatment of Au nanoparticles up to 700 °C, on SiO2 , let particles grow from 4 to 6 nm while on TiO2 from 3 to 13 nm. Also, Al_2_O_3_ provides a nonreducible support and a nonoxidizing atmosphere for stable supported Au nanoparticle [33]. To the best of our knowledge we obtained lowest effective nobel metal loading as compared to previous reports [33–36].

In the present work, a set of Au/γ -Al_2_O_3_ catalysts were prepared by successive impregnation method. For this purpose, γ -Al_2_O_3_ support in granular shape (mesh size = 0.6-1 mm and BET surface area = 175 m^2^g^-1^ ) was prepared by sol gel route. All the prepared samples were characterized by XRD, surface area analysis, SEM, and FTIR. The aim of this work was to synthesize cost efficient supported gold catalysts with low noble content and evaluate their catalytic activity for the reduction of 4NP to 4AP using SBH as a reducing agent by UV-visible spectroscopy. Interestingly, the prepared catalysts showed excellent activity towards the conversion 4-NP to 4AP, owing to good metal dispersion and metal-support interactions.

## 2. Experimental

### 2.1. Materials

Aluminium chloride hexahydrate (AlCl_3_ .6H_2_O, Merck, 99.9%), ammonium hydroxide (NH_4_OH, Fisher, 33%), and gold chloride trihydrate (HAuCl_4_ .3H_2_O, Merck, 99.9%) were used as precursors. Distilled water with the ionic conductivity ≤10 μS cm^-1^ was used in the preparation of all aqueous solutions.

### 2.2. Preparation of supported catalysts

Alumina support was produced by sol gel method using 0.4M AlCl_3_ .6H_2_O solution and 0.4M NH_4_OH solution. The initial pH of NH_4_OH solution was recorded as 12. AlCl_3_ .6H_2_O solution and was added to NH_4_OH solution slowly with continuous stirring until alumina sol was obtained at pH 8. Alumina sol was aged in heating oven at 80 °C for 48 h. Under vacuum filtration gel was washed with deionized water to remove the impurities (i.e. Cl^-^, Na^+^, NH^+^_4_ etc.). If these impurities remain in the support, they can affect the efficiency of catalyst. Alumina gel was converted to the granules by a method described earlier [37–38]. In order to obtain gamma phase, granules were heat treated in programmable muffle furnace up to 750 °C for 16 h. Granular support (γ -Al_2_O_3_) was obtained having mesh size, between 0.6 and 1 mm and BET surface area of 175 m^2^g^-1^ .

A series of Au/γ -Al_2_O_3_ catalysts were prepared by wet impregnation method with composition of 0.2%, 0.4%, 0.6%, 0.8%, 1% by weight. Impregnation was accomplished on predried γ -Al_2_O_3_ granular support using required volume of HAuCl_4_ precursor. All catalysts were soaked overnight, dried in oven at 120 °C for 2 h and further calcined at 550 °C for 3 h. Finally, catalysts were reduced in H2 gas (99.99 %) flow at 550 °C for 3 h.

### 2.3. Characterization

X-ray diffraction (XRD) patterns of the synthesized materials packed in an aluminium glass holder were recorded at room temperature using Philips PW-1840 diffractometer with CuKα radiation (λ = 0.154 nm) in 2θ range of 20°–80°with scan rate of 0.01 θ s^-1^.

Surface area analysis (SAA) of supported catalyst was carried out on KELVIN 1042. Degassing of the samples was carried out at 250 °C with N_2_ as carrier gas. BET surface area was obtained from nitrogen adsorption–desorption isotherms measured at liquid nitrogen temperature (–196 °C). Pore size and total pore volume were obtained by employing BJH method.

Fourier transform infrared (FTIR) spectroscopy analysis was carried out on Thermo Scientific, model 6700FTIR made of USA and the technique applied was ATR (attenuated total reflectance) at a range of 400– 4000cm^-1^ . Scanning electron microscopy (SEM) was used to examine surface morphology of the samples on JEOL-JED 2300 instrument and on TEM Philips TEM CM12. A UV-visible spectrophotometer (UV-1601 Shimadzu spectrophotometer) with wavelength range of 190–1100 nm was utilized to collect spectral data.

### 2.4. Catalytic activity test

The catalytic performance of Au/γ -Al_2_O_3_ catalysts was evaluated with the help of a well-studied reaction of conversion of 4NP to 4AP using SBH as a reducing agent and UV-visible spectra were recorded during the reaction [32]. In a typical reaction, 5 mg of sample in a standard quartz cuvette was taken and then 100 μL of 1mM 4NP solution was added. The volume was adjusted to 3.5 mL with water and finally ice-cold SBH (100 μL, 100 mM) was added to the above quartz cuvette. The decrease in absorbance of 4NP was recorded with a time interval of 3–5 min (according to the reaction conditions). Rate constants were observed to be dependent upon the wt% of Au nanocatalyst and temperature. The catalytic reaction was carried out at various temperatures to calculate kinetic parameters (rate constant and activation energy).

## 3. Results and discussion

### 3.1. Characterization of support and catalysts

**Figure 1:**
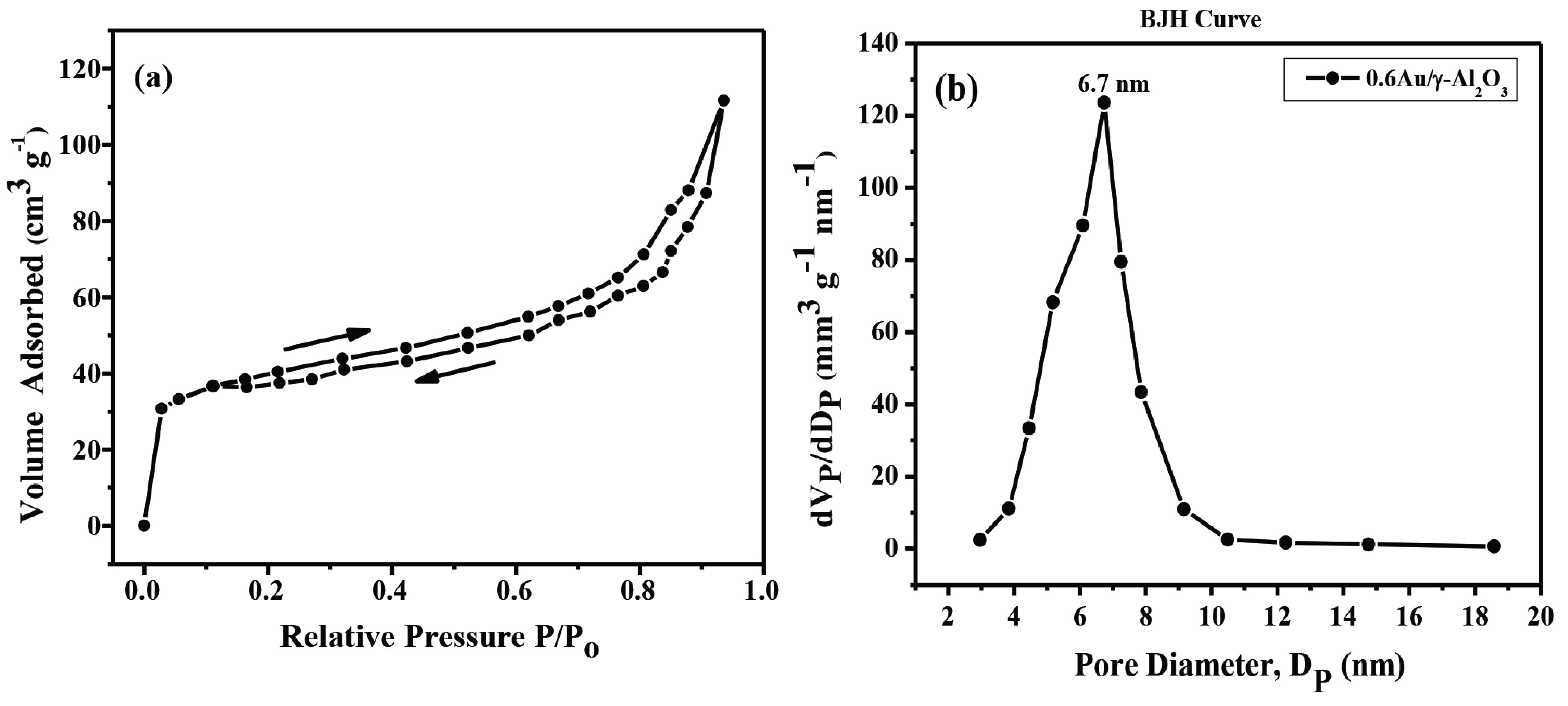
Adsorption-desorption isotherms (a) and pore size distribution of 0.6Au/γ -Al_2_O_3_ catalyst using BJH method (b).

The nitrogen adsorption-desorption isotherms (Figure 1a) of 0.6Au/γ -Al_2_O_3_ sample with type IV shape designated the presence of mesopores with uniform pore size distribution [37, 38]. Table 1 presents the composition, BET surface area, pore volume, average pore diameter, and crystallite sizes of γ -Al_2_O_3_ support and Au/γ -Al_2_O_3_ catalysts. Figure 1b shows unimodal distribution of pore for Au/γ -Al_2_O_3_ catalyst which exhibited with maxima centred at ~7 nm. BET surface area and pore volume of Au catalysts were found to decrease with respect to γ -Al_2_O_3_ support. It can be correlated to occlusion of alumina pores with Au particles during impregnation process. In addition, with further increase in metal content, BET surface area of the catalysts was examined to decrease which might be due to metal-on-metal deposition besides the blockage of alumina pores with metal. After the deposition of gold NPs on γ -Al_2_O_3_ support the surface area of the support decreased from 175 m^2^g^-1^ to 129 m^2^g^-1^ and pore volume decreased from 0.47 cm^3^g^-1^ to 0.310 cm^3^g^-1^ . This reduction in surface area and pore volume of the pure support after metal loadings provides an evidence of the successful deposition of the gold NPs by impregnation method.

**Table 1:** Table 1 presents the composition, BET surface area, pore volume, average pore diameter, and crystallite sizes of γ -Al_2_O_3_ support and Au/γ -Al_2_O_3_ catalysts.

Samples	Composition	Surface properties		
	Au^a^(wt%)	S^b^_A_ (m^2^g^-1^)	V^C^_P_ (cm^3^g^-1^)	D^d^_p_ (nm)
γ-Al_2_O_3_	0	175.0	0.47	7.8
0.2Au/γ-Al_2_O_3_	0.2	165.3	0.45	6.9
0.4Au/γ-Al_2_O_3_	0.4	155.1	0.43	6.8
0.6Au/γ-Al_2_O_3_	0.6	140.5	0.38	6.7
0.8Au/γ-Al_2_O_3_	0.8	135.8	0.35	6.3
1Au/γ-Al_2_O_3_	1	129.2	0.31	6.0

^a^Nominal Au metal content, ^b^BET specific surface area, ^c^Pore volume, ^d^Pore diameter determined by surface area analyser.

**Figure 2:**
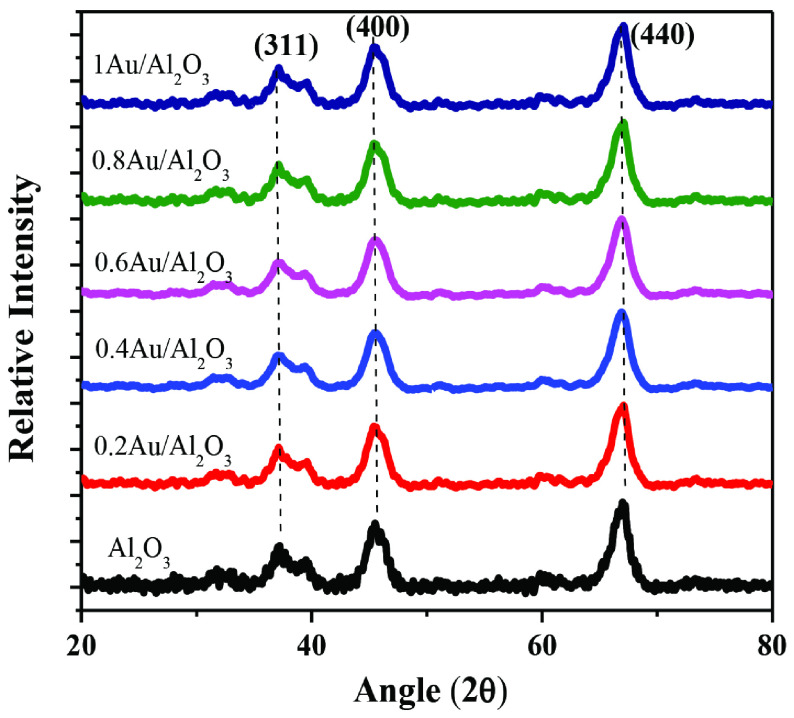
XRD patterns of γ -Al2O3 support and Au/γ -Al2O3 catalysts.

Figure 2 shows XRD patterns of Al_2_O_3_ support and Au/γ -Al_2_O_3_ catalysts. The significant peaks in XRD pattern of γ -Al_2_O_3_ were appeared at 2θ ~37°, 46°, and 66° having (311), (400), and (440) hkl values, respectively. These values matched with standard ICDD Card No 00-001-1303 thus indicating the formation of cubic and pure gamma phase Al_2_O_3_ NPs. For all Au catalysts, no additional XRD peaks were observed besides the significant peaks of γ -Al_2_O_3_ thus showing either complete incorporation of Au NPs into γ -Al_2_O_3_ pores or metal particles are too small which could not be detected by XRD. The crystallite sizes (DXRD) of Au/γ -Al_2_O_3_ catalysts were attained by Debye Scherrer formula:

DXRD (nm) = Kλ/β cosθ ,

where K is particle shape factor (0.9 for cubic system), λ is wavelength of X-ray beam (0.154 nm), β is peak broadening (FWHM), and θ is diffraction angle.

DXRD values of all samples were calculated by using high intensity diffraction peak and were found ~6 nm.

**Figure 3:**
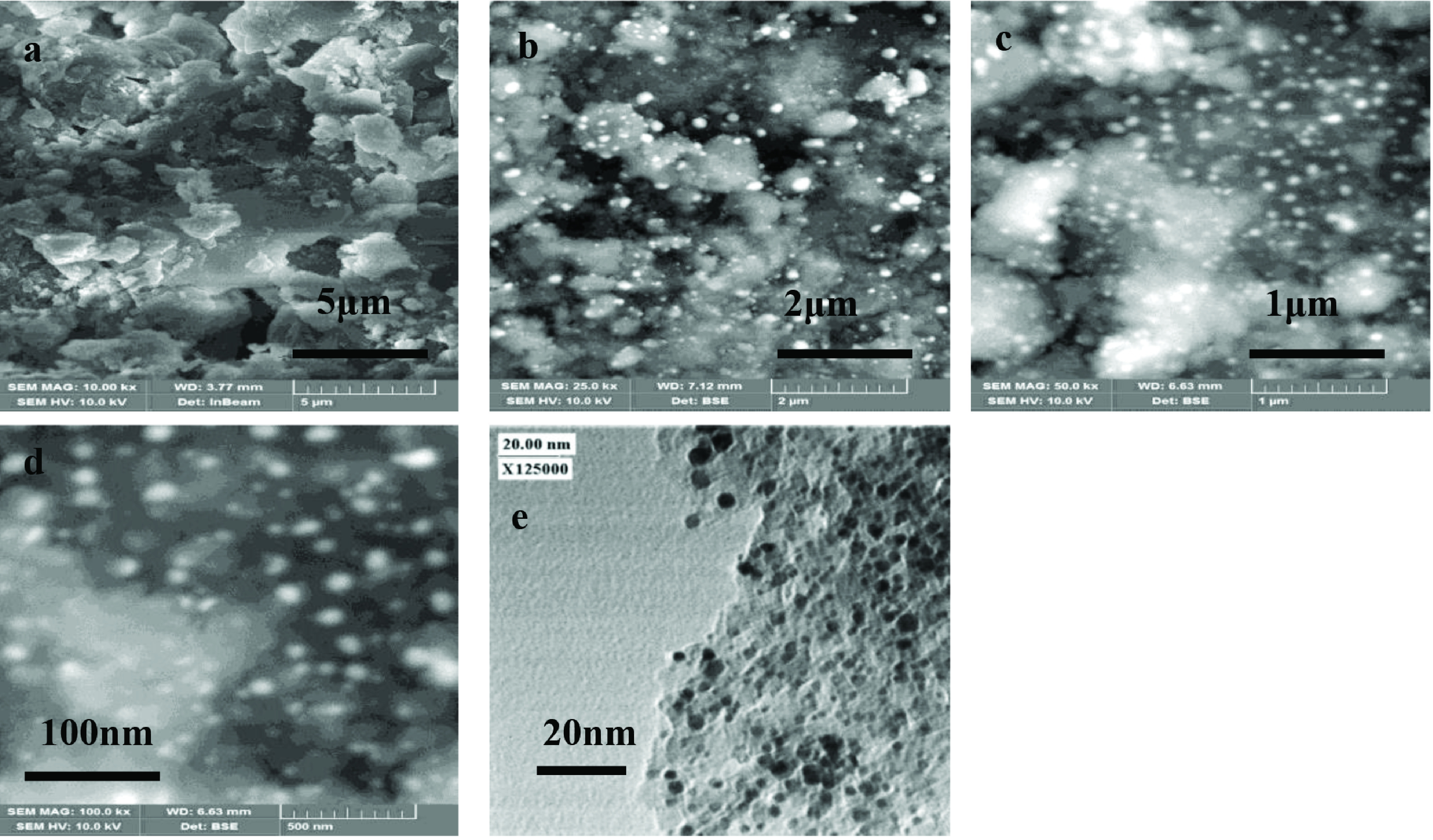
SEM images of synthesized γ -Al_2_O_3_ support (a), 0.6Au/ γ -Al_2_O_3_ catalyst (b-d), TEM image of 0.6 wt% Au/γ -Al2O3 catalyst (e).

To investigate the morphology of synthesized materials, SEM images were taken. Figure 3a presents SEM image of γ -Al_2_O_3_ support calcined at 750 °C. With a coarse surface, γ -Al_2_O_3_ was observed having particles of irregular size and shape. In contrast, 0.6Au/γ -Al_2_O_3_ catalyst (Figure 3b-3d) presented homogenous distribution of Au NPs over the surface of γ -Al_2_O_3_ support at different resolutions. Au particles were found spherical in shape having uniform size. Here, high metal dispersion of this optimal composition was corresponded for its excellent activity towards conversion of 4NP to 4AP. Figure 3e shows TEM image of 0.6Au/γ -Al_2_O_3_ showing AuNPs of ~6 ±2 nm.

**Figure 4:**
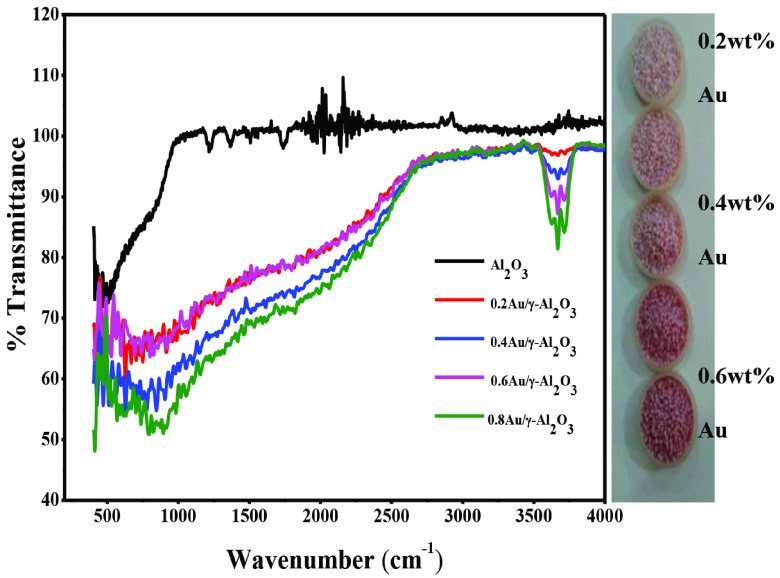
FTIR spectra of γ -Al2O3 and supported Au catalysts.

FTIR spectra of pure γ -Al_2_O_3_ along with Au supported analogues are presented in Figure 4. In pure γ -Al_2_O_3_ the absorption band around 3400 cm^-1^ can be ascribed to stretching mode of hydroxyl (OH) group. The peak at 1640 cm^-1^ may be attributed to the bending vibration of weakly bound molecular water. The absorption bands in the region of 600–660cm^-1^ may be attributed to the stretching mode of AlO4 and AlO6 while absorption bands below 600 cm^-1^ appeared due to the bending modes of vibrations of AlO6 groups. In the case of Au/γ -Al_2_O_3_ catalysts vibrational and stretching modes were affected by Au content in alumina. Au being paramagnetic in character may interfere with electron density on Al = O bond resulting in dilution of electron density on doubly bonded oxygen and reduction in the rigid character of Al = O bond in Al_2_O_3_ . As a consequence, the peak was shifted towards higher wave number i.e. from 600 to 700 cm^-1^ . FTIR, being a very sensitive technique, can detect even small amount of impurity present in the samples. This fact is evident when we compare the FTIR and XRD results in present case, where we did not get new diffraction peaks after depositing gold on alumina support. On the other hand, changes in the FTIR spectra pattern of pure alumina after gold loading were detected. The change in the colour of the Au/γ -Al_2_O_3_ catalyst with increase in concentration of the deposited gold is evident in the Figure 4. From 0.2wt% loading of Au to 1wt% loading of Au metal content, Au/ γ -Al_2_O_3_ supported catalyst show a variation from light pink to a reddish pink coloration. This physical change in the colour of Au/γ -Al_2_O_3_ catalysts is because of the well-known coupling of the Plasmon responses of the attached nanoparticles. As the coverage increases, the average interparticle separation decreases and the above-mentioned coupling increases.

### 3.2. Catalytic testing of prepared catalysts

**Figure 5:**
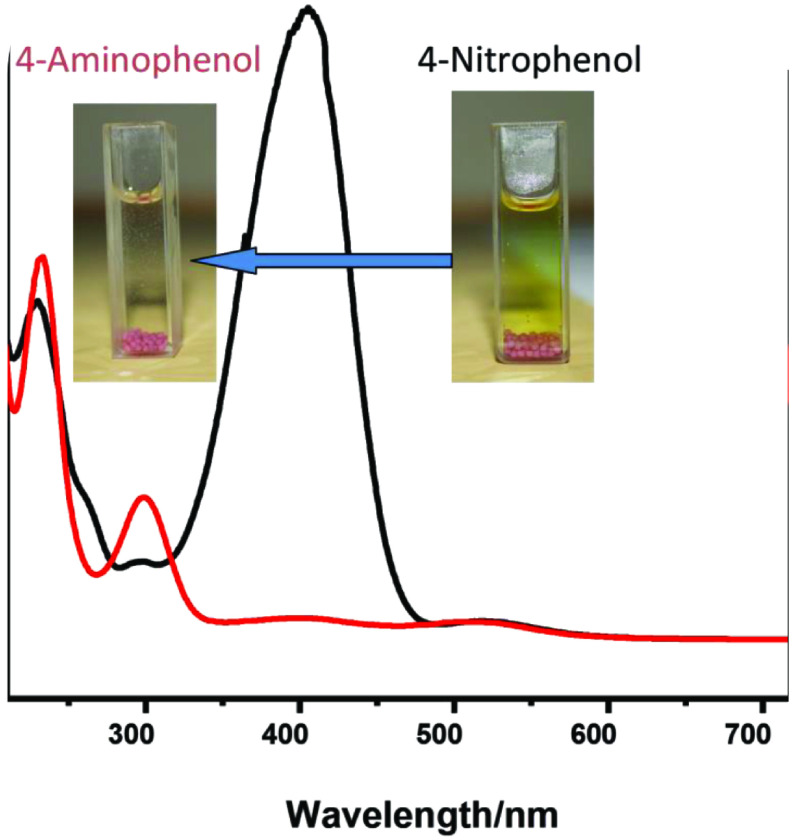
UV-visible spectra for the transformation of 4-nitrophenol to 4-aminophenol over 0.6Au/ γ -Al_2_O_3_ catalyst.

Figure 5 shows the typical UV-visible spectra for the conversion of 4NP to 4AP. A significant decrease in peak intensity was observed upon the conversion. In the spectrum 4- nitrophenol was depicted λmax at ~400 nm and was shifted to ~295 nm during the catalytic reaction thus confirming the formation of 4-aminophenol. The solution (4-Nitrphenol) is light yellow in colour, while 4-aminophenol is colourless. The physical change from light yellow colour to a colourless solution was also examined during the reaction, depicting a complete transformation from 4-nitrophenol to 4-aminophenol. We selected 0.6wt% Au/γ -Al_2_O_3_ for complete catalytic testing. The reason for selecting this wt% is its ability to catalyse reaction at reduced time as compared to lower (0.2 and 0.4wt%) and higher (0.8 and 1wt%) values. Lower wt% has less deposition of gold nanoparticles and higher wt% block the mesopores in γ -Al_2_O_3_ , which in turn decreases the availability of active sites of nanogold. Thus, out of the 5 prepared catalysts, 0.6Au/ γ -Al_2_O_3_ was chosen to proceed for further studies.

**Figure 6:**
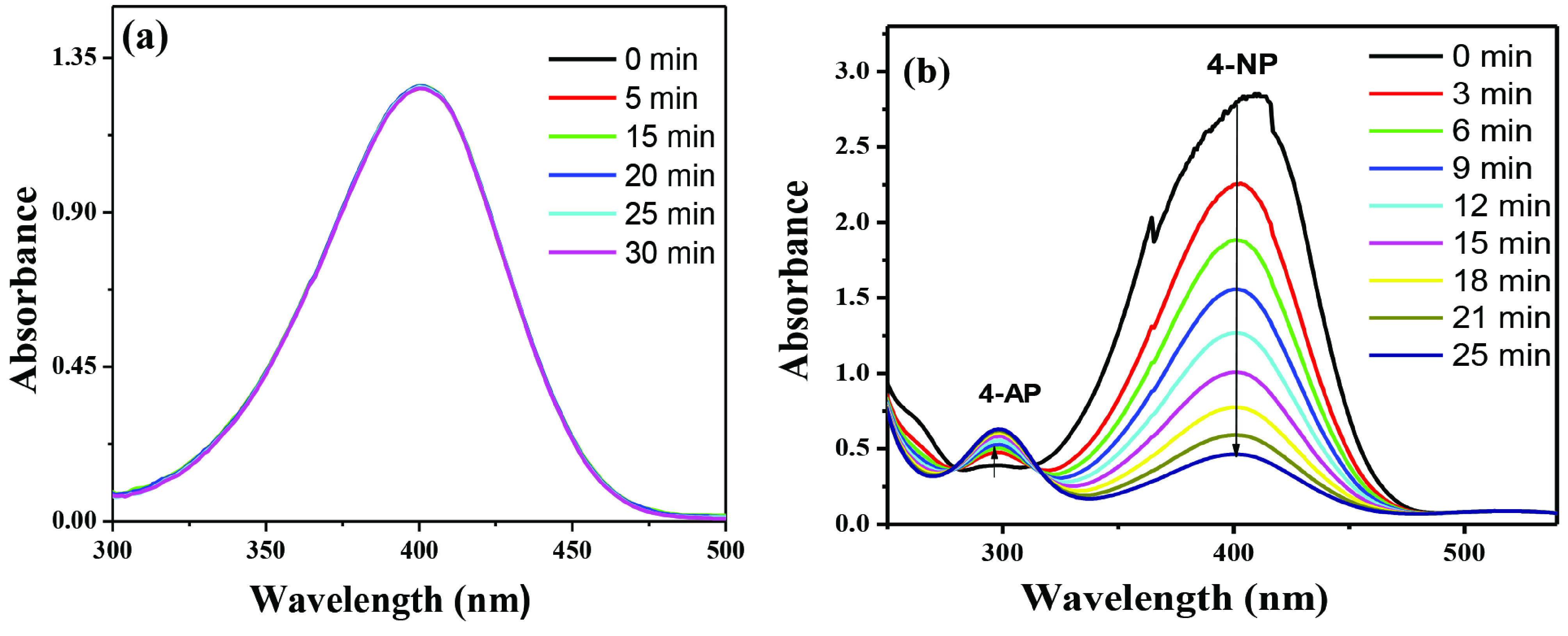
UV-visible spectra for the catalytic activity of γ -Al_2_O_3_ at different time intervals (a) and 0.6Au/ γ -Al_2_O_3_ catalyst at 25 °C(b).

Figure 6a presents that γ -Al_2_O_3_ support has no ability to catalyse the reaction at room temperature during different time intervals and even in the entire temperature range studied (higher temperature data is not shown). In the case of 0.6Au/γ -Al_2_O_3_ catalyst, a significant change in UV-visible absorbance was observed (Figure 6b) for conversion reaction thus depicting the presence of active sites on the surface of 0.6Au/γ - Al_2_O_3_ catalyst.

**Figure 7:**
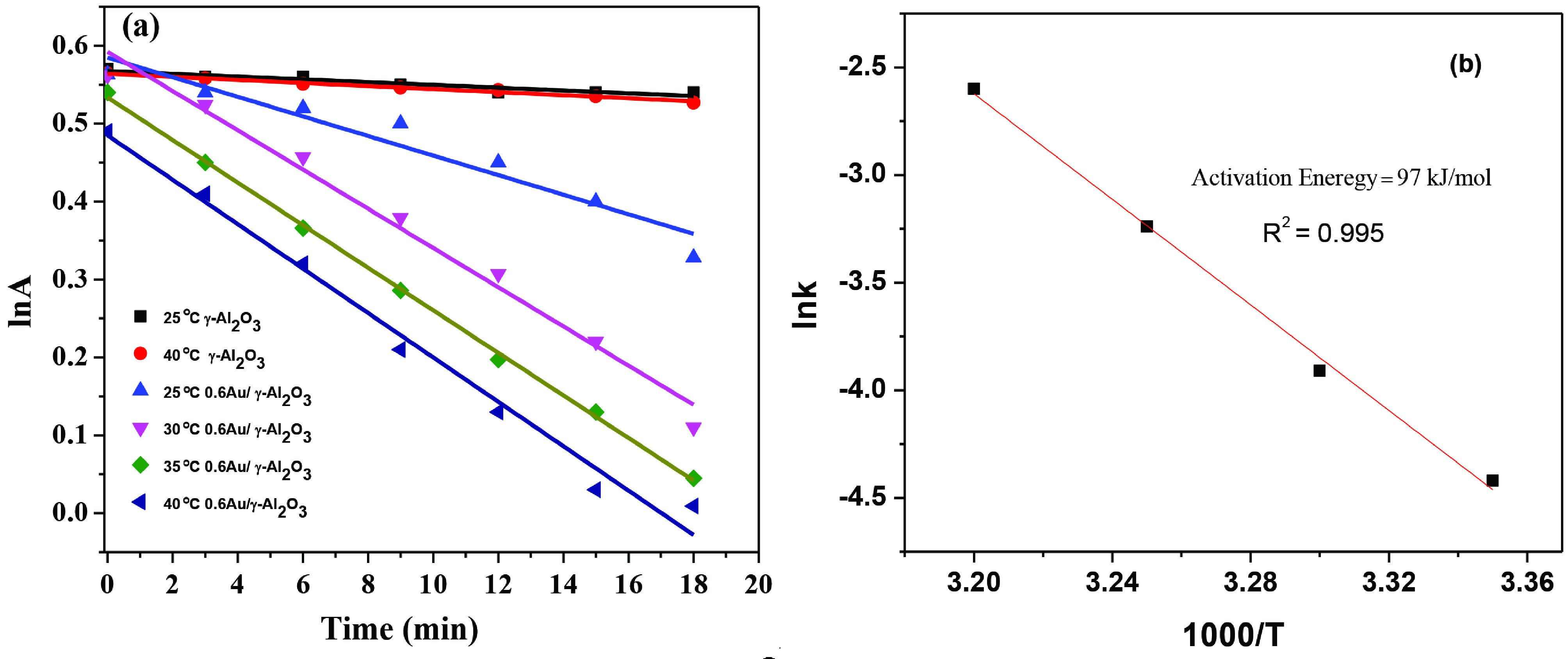
lnA vs t plot for the conversion reaction over γ -Al_2_O_3_ support and prepared Au/γ -Al_2_O_3_ catalysts (a) and Arrhenius plot for the activity of 0.6Au/γ -Al_2_O_3_ catalyst at different temperatures(b).

Temperature was found to have a pertinent effect on the catalytic performance. The catalysed reaction showed change in its kinetics on increasing the temperature from 25 °C to 40 °C as can be seen in Figure 7a The rate constant was calculated using the following equation:

lnA/A°= –kt Eq (1)

lnA-ln A°= –kt Eq (2)

ln A= ln A° –kt Eq (3)

Where, A is absorbance of 4-NP at time t, A°is initial absorbance of 4-NP, k is the rate constant, and t is the time interval for UV-visible data collection.

The plot of ln A versus t (Figure 7a) at different temperatures produces a straight line with the slope equivalent to rate constant.

Table 2 presents the rate constant values of the reaction catalysed by Au/γ -Al_2_O_3_ catalyst at different temperatures.

The rate constant value was observed to increase 5.4 times on increasing the temperature from 25 °C to 40 °C. The activation energy (Ea) was calculated using Arrhenius equation.

k = A exp (-Ea /RT) Eq (4)

**Table 2:** Kinetic parameters of Au/γ -Al_2_O_3_ catalyst.

Sample	Temperature (°C)	k ×10^-4^(min^-1^)	Ea (kJmol^-1^)
0.6Au/γ-Al_2_O _3_	25	1.2 ±1 × 10^-5^	97 ±14
	30	2.0 ±3 ×10^-5^	
	35	3.9 ±2 ×10^-5^	
	40	7.4±5 × 10^-5^	

Figure 7b shows the Arrhenius plot of rate constant versus the reciprocal of temperature (both in logarithmic scale) with a straight line. The activation energy was found to be 97 Kjmol^-1^. This value of Ea is high as compared to the E_a_ for free colloidal suspension of gold nanoparticles as given in our previous results .[31,39]. Although gold NPs deposited on γ -Al_2_O_3_ were well dispersed as can be seen in Figures 3b-3e, still all the active sites of these supported gold NPs were not available for catalysis. Since we see how the physical state (colloidal or supported) of a catalyst affects its catalytic performance, we can conclude that size and physical state of the catalyst play a very crucial role in their catalytic performance.

**Figure 8:**
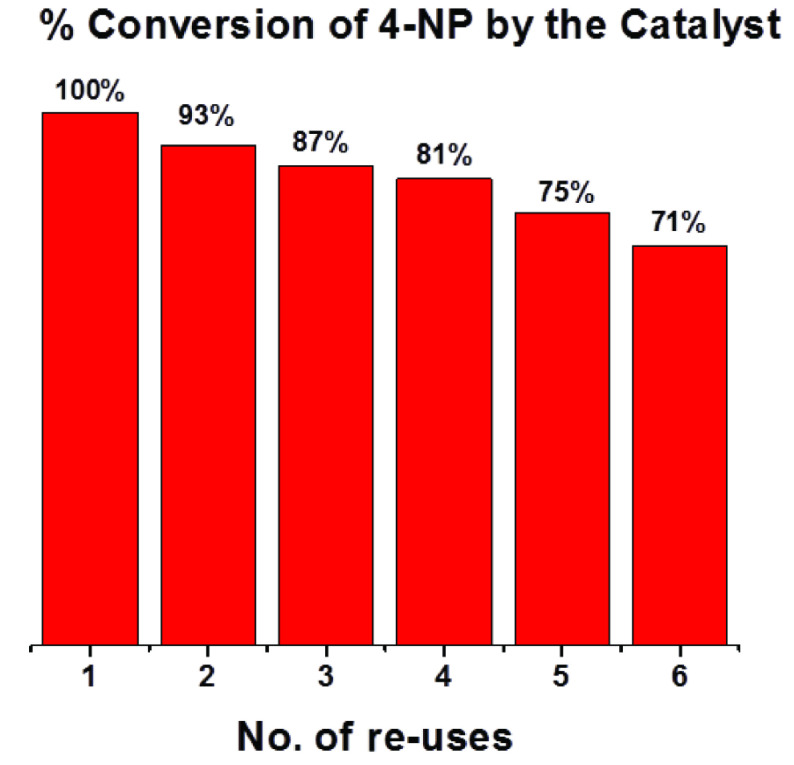
Recyclability of 0.6Au/γ -Al2O3 catalyst over many uses.

Supported gold catalyst was well stable during the course of reaction and even after many cycles in comparison to the reported colloidal alloy nanoparticles [39], which show aggregation during and after the catalysed reaction. It is reported that capping material is usually stripped off during the catalytic reaction, so this may contribute towards the nanocatalyst aggregation which is not observed in the present case for the supported catalyst. Present catalyst is also recyclable, it was used 6 times with appreciable efficiency (Figure 8). The AuNPs supported on the surface of mesoporous γ -Al_2_O_3_ were found to be stable and did not leach out into the solution after heating at 60 °C for 1h. This was confirmed by taking UV-visible of solution which shows no SPR for free AuNPs or gold salt, also SEM imaging, which showed no free AuNPs in the solution (Figure 3e).

**Figure 9:**
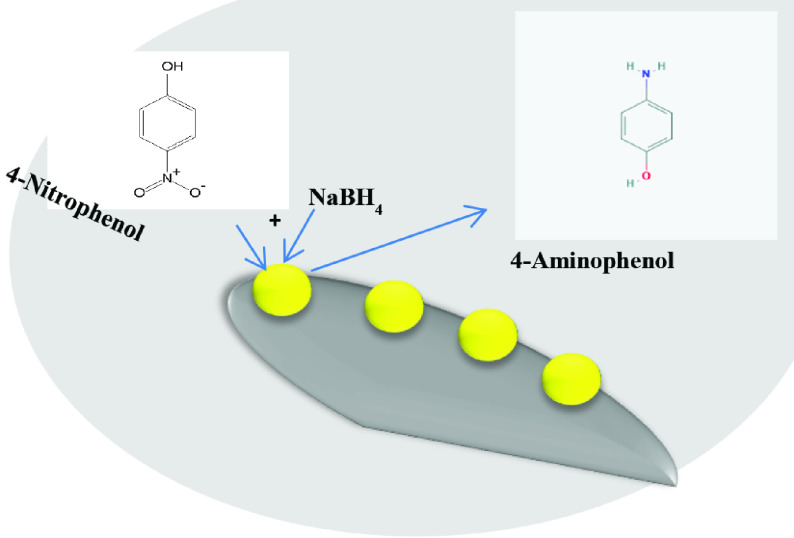
Mechanism of 4-NP reduction on the surface of 0.6Au/γ -Al_2_O_3_ catalyst following Langmuir Hinshelwood model where both 4-NP and NaBH4 adsorb at the surface of nanocatalyst and 4-AP as a product desorb.

The dependence of the reduction of 4-NP by BH as a function of temperature can be modelled in terms of Langmuir-Hinshelwood model [40–42]. According to this model both reactants must be adsorbed to the surface of the nanocatalyst to react and this reaction was found to be kinetics controlled where the transportation of the reactants through the solution was not the rate determining step, rather the formation of 4-AP was the rate determining step. The adsorbed species can then react, and the product will desorb from the surface of supported gold catalyst (Figure 9). Comparative performance of Au/γ -Al_2_O_3_ catalyst with available literature is compiled in Table 3.

**Table 3:** Comparison of wt% Au loading and catalytic activity of our work with literature.

Sample code	Reaction studied	Wt% Au loading	Rate constant (min^-1^)	Year	Reference
Au/Al_2_O_3_	[Fe(CN)_6_]^3-^ with (S_2_O_3_)^2--^	3.6	0.0017	2012	19
Au/Starch	4-Nitrophenol reduction with NaBH_4_	4.32	0.0081	2017	36
Au/Carbon	4-Nitrophenol reduction with NaBH_4_	2	0.660	2019	35
Au/Al_2_O_3_	Thermal stability	4	-	2019	33
Au/Al_2_O_3_	4-Nitrophenol reduction with NaBH_4_	0.6	0.00012	2020	This work

## 4. Conclusions

In the present work, a sol–gel method was utilized to synthesize mesoporous γ -Al_2_O_3_ in granular shape with BET surface area of 175 m^2^g^-1^ , which was reduced to 125 m^2^g^-1^ after gold deposition. Au/γ -Al_2_O_3_ catalysts with 0.2–1wt% metal contents were prepared via impregnation method. The results of SAA indicated a decrease in BET specific surface area of Au catalysts due to occlusion of γ -Al_2_O_3_ pores with metal particles while pore diameter increased up to 8 nm at the expense of sintering process. XRD results depict the formation of single phase γ -Al_2_O_3_ NPs. SEM depicted a uniform dispersion of Au particles on the surface of γ -Al_2_O_3_ support. The metal particles were found spherical. The catalytic activity of Au/γ -Al_2_O_3_ (0.6wt%) catalyst was evaluated for reduction of 4NP to 4AP at 4 temperatures i.e. 25, 30, 35, and 40 °C and UV-visible spectra were recorded. γ -Al_2_O_3_ support exhibited no activity for the conversion reaction while Au catalyst showed a significant change in UV-visible absorbance with rate constant 1.2 × 10−4 min−1 . Use of low noble metal content made the process cost effective and efficient at the same time.
